# Supplementary material for: Anti-Parkinson Effects of Holothuria leucospilota-Derived Palmitic Acid in Caenorhabditis elegans Model of Parkinson’s Disease
Source: Mar Drugs. 2023 Feb 23;21(3):141. doi: 10.3390/md21030141 (PMC10051922; doi:10.3390/md21030141)
Supplement: Supplementary file 1 [file marinedrugs-21-00141-s001.zip › marinedrugs-2214536-supplementary.pdf]

Supplementary data for Manuscript entitle

“Anti-Parkinson effects of *Holothuria leucospilota*-derived palmitic acid in *Caenorhabditis elegans* model of Parkinson’s disease”

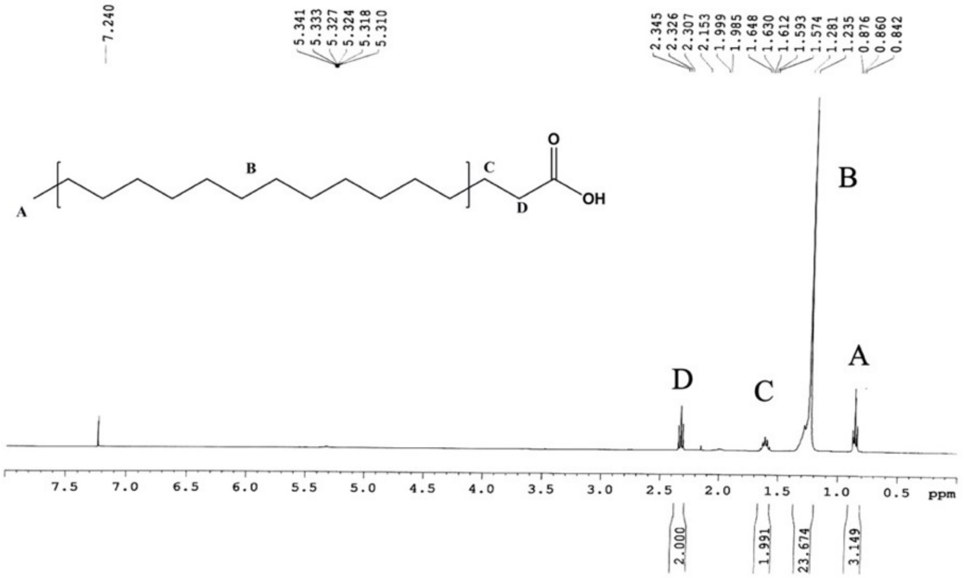

Figure S1. ^{1}H-NMR spectrum of HLEA-P3, palmitic acid or hexadecanoic acid, in $CDCl_3$.

Mass Spectrum SmartFormula Report

Analysis Info

Analysis Name	D:\Data\Apichart\ESI\AS-HRMS 1012 (pos).d	Acquisition Date	7/3/2022 5:21:23 PM
Method	tune_low.m	Operator	RU
Sample Name	EA3	Instrument	micrOTOF 8213750.10411
Comment			

Acquisition Parameter

Source Type	ESI	Ion Polarity	Positive	Set Nebulizer	0.3 Bar
Focus	Not active			Set Dry Heater	180 °C
Scan Begin	50 m/z	Set Capillary	4500 V	Set Dry Gas	4.0 l/min
Scan End	2000 m/z	Set End Plate Offset	-500 V	Set Divert Valve	Waste

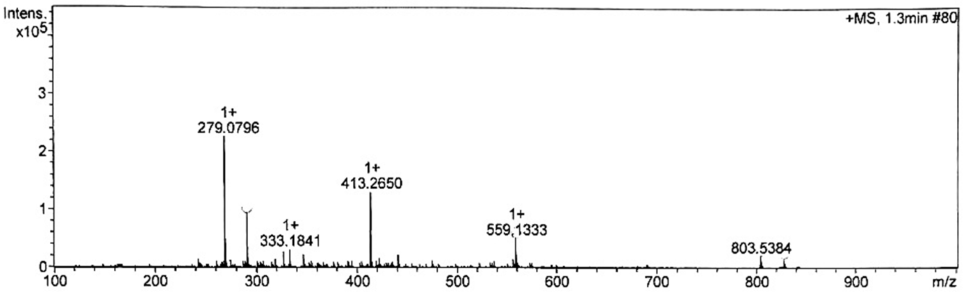

Meas. m/z	#	Ion Formula	m/z	|err| [mDa]	err [ppm]	Mean err [ppm]	rdb	N-Rule	e⁻ Conf	mSigma
279.079646	1	C15H10N4Na	279.079767	0.1	0.4	0.1	12.5	ok	even	7.9
	2	C14H14NaO4	279.078430	1.2	-4.5	-4.5	7.5	ok	even	12.8
	3	C16H32NaO2	279.075744	3.9	14.5	-15.2	8.5	ok	even	26.4
291.061360	1	C16H12NaO4	291.062780	1.4	-4.9	3.1	10.5	ok	even	13.3
	2	C17H8N4Na	291.064117	2.8	9.5	7.4	15.5	ok	even	19.9
	3	C12H8N6NaO2	291.060094	1.3	-4.4	-6.7	11.5	ok	even	22.6
	4	C11H12N2NaO6	291.058757	2.6	8.9	-11.0	6.5	ok	even	31.3
559.133254	1	C32H24NaO8	559.136338	3.1	-5.5	4.0	20.5	ok	even	9.5
	2	C29H16N10NaO2	559.134991	1.7	3.1	1.2	26.5	ok	even	13.6
	3	C28H20N6NaO6	559.133653	0.4	-0.7	-1.0	21.5	ok	even	15.5
	4	C25H12N16Na	559.132305	0.9	1.7	-3.9	27.5	ok	even	20.0
	5	C27H24N2NaO10	559.132316	0.9	1.7	-3.3	16.5	ok	even	23.7
	6	C24H16N12NaO4	559.130968	2.3	-4.1	-6.1	22.5	ok	even	26.6
	7	C39H20NaO3	559.130465	2.8	-5.0	-6.5	29.5	ok	even	40.5

Figure S2. HRMS spectrum of HLEA-P3, palmitic acid, in ES, positive ion mode
